# Notes and News

**Published:** 1891-06-06

**Authors:** 


### Notes and News.

COLLECTED AND COMMUNICATED.

On May the 28th Princess Louise paid her promised visit to the Metropolitan Hospital in Kingsland Road. She arrived about four o'clock, accompanied by Mr. Cyril Flower, M.P., and was received at the hospital by the Bishop of Bedford, Mr. Joseph Fry, chairman of the Committee of Management, Sir James Paget, Rev. R. S. Hassard, Lieut.-Col. Montefiore, Dr. Drysdale, and Mr. F. D. Mocatta. A bouquet was presented by a little girl, a patient in the hospital, and the Princess then went through the various wards of the hospital, and before leaving, readily consented to the large women's ward being named "Louise Ward," in commemoration of her visit.

A quarterly general court of the Governors of the Middlesex Hospital was held on the 28th ult. Mr. Robert Ruthven Pym was in the chair, and a large number of Governors were present. After some formal business had been transacted, the Court decided to authorise the expenditure of £1,300 for the provision of new teak floors for seven of the wards, and also sanctioned the sale of £5,000 stock to meet the current expenses of the Hospital, and the sale of £45,000 consols (forming part of the endowment of the Hospital) for re-investment in stocks bearing a higher rate of interest. The proceedings terminated with a vote of thanks to the Chairman.

The Earl of Strafford presided on May 29th at the twenty-fourth annual Court of the Governors of the East London Hospital, Shadwell. The report stated that in the total number of patients under medical and surgical treatment at the Hospital there was an increase of 2,575 over the number for the previous year. The number of attendances on out-patients and casualty cases was 32,223. The large increase in the number of adult casualty cases had so greatly overtaxed the capacity of the surgical staff that attention had to be restricted to only the most urgent cases. This excellent little hospital deserves wider support, especially in the way of annual subscriptions and of donations to the building fund.

We regret to announce the death of Dr. Samuel Crompton, at the age of seventy-four, which took place at Cranleigh, Surrey. He received his medical education partly at the Manchester School of Medicine, and afterwards at St. Bartholomew's Hospital, where he took honours. He was subsequently elected a member of the Royal College of Surgeons, London, and received his M.D. from St. Andrew's. His connection with Henshaw's Blind Asylum, Old Trafford, is the most prominent part of his life, from a public point of view. He made blindness a special study, and published, in 1849, a treatise, entitled "Results of an Investigation into the Causes of Blindness." Dr. Crompton was the grandson of Samuel Crompton, the inventor of the spinning-mule.

An interesting meeting of the Burial Reform Association was held at Grosvenor House on Monday, the Duke of Westminster in the chair. Several doctors were present, and the question daily grows a more important one from the health standpoint.

At the 39th annual court of the Governors of the Hospital for Sick Children, Great Ormond Street, the report of the Committee of Management contradicted the prevalent rumour that the hospital has been placed in a condition of independence by the recent bequest of Mr. Barry. That bequest will produce an annual income of about £1,400, but the Committee will still be obliged to raise, by donations and subscriptions, not less than £8,000 a year.

A new Cottage Hospital at Blanford was opened by the Hon. Miss Portman, in April last. The building contains five wards, and is most conveniently arranged, the wards for men forming one wing, and those for women the other; while between the two there is a committee or convalescent room. The building is heated throughout by hot-water pipes, and the sanitary arrangements are perfect. Lord Portman has endowed a bed in the women's ward, at £50 a-year, whilst in the men's ward there is a similar gift for one year from the new mission-hall. The furnishing and arrangements reflect much credit upon the Committee of Management, and Miss Short, the Matron.

The new General Infirmary of Derby will consist of thirteen blocks of buildings. The central block, round which the wards and other buildings are grouped, will contain the secretary's office, waiting room, room for medical staff, and the casualty department, and above these the board room, the residential quarters for the medical staff and Matron, and servants' bedrooms. This building will be connected with another block, to be called the Back Administration, by a corridor, which will also cut at right angles the main corridor communicating with the five ward pavilions and the out-patient department. Iron, bedded in concrete, will enter largely into the construction, and the floors are to be of teak. Externally, the buildings will be faced with red brick with stone mullions, cornices, and mouldings. The laundry is to be contained in a separate block with a separate wash-house for infected linen. The remaining four blocks will be devoted respectively to the chapel, operation room, nurses' home, and mortuary, the last containing also a post-mortem room, and ambulance house and stable. The architects are Messrs. Young and Hall.

At a meeting of the City Commission of Sewers, at which Mr. Sheppard Scott presided, the report of the Medical Officer of Health (Dr. Sedgwick Saunders), relative to the sanitary condition of St. Bartholomew's hospital, was brought up for consideration. The report recommends the ordering of the demolition and the removal of the whole of the drainage of the hospital, and the directing that a more reasonable and safer system be substituted and constructed to their satisfaction. Dr. Saunders, replying to questions, said that the various improvements instituted by the hospital authorities were excellent as far as they went, but they stopped at the great question of what should be done with the main drainage. Those huge unnecessary sewers would have sufficed for a population of three or four million people, while they were dealing with 1,000. He suggested three pipes of fifteen inches, which would be ample. The works might be done in three sections, and he staked his professional reputation that they might be effected without closing the hospital for a single day. After a discussion on various details in the report, it was unanimously resolved, on the motion of Mr. Woodrow, that the orders suggested by the Medical Officer should be served on the hospital authorities.

